# Supplementary material for: COVID-19-Induced Acute Respiratory Distress Syndrome Treated with Hyperbaric Oxygen: Interim Safety Report from a Randomized Clinical Trial (COVID-19-HBO)
Source: J Clin Med. 2023 Jul 24;12(14):4850. doi: 10.3390/jcm12144850 (PMC10381696; doi:10.3390/jcm12144850)
Supplement: Supplementary file 1 [file jcm-12-04850-s001.zip › jcm-2512027-supplementary.pdf]

Table S1. Overview of Adverse Events

	Group					
			Best practice		Hyperbaric oxygen (HBO)	
	Count	Sum	Count	Sum	Count	Sum
AEFREQ	1	1	13	55	14	40

Table S2. Summary of Adverse Events by System Organ Class and Preferred Term

		Group	
		Best practice	Hyperbaric oxygen (HBO)
	nt	Count	Count
SOC			
Cardiac disorder	0	2	0
Cardiac disorders	0	1	3
Ear and labyrinth disorders	0	1	1
General disorders and administration site conditions	0	0	1
Infections and infestaions	0	1	0
Infections and infestations	0	1	1
Metabolism and nutrition disorders	0	2	0
Nervous system disorders	0	0	1
Psychiatric disorders	0	1	2
Respiratory, thoracic and mediastinal disorders	0	3	4
Vascular disorders	0	1	1
PT			
Abscess	0	1	0
Anxiety	0	1	0
Atrial fibrillation	0	2	0
Bacteremia	0	1	0
Bradycardia	0	1	0
Cardiogenic Shock	0	0	1
Chest pain	0	0	1
Claustrophobia	0	0	1

Cough	0	1	0
Depression	0	0	1
Diabetes mellitus	0	2	0
Ear pain	0	1	1
Fever	0	0	1
Fungal infection	0	0	1
Headache	0	0	1
Hypotension	0	1	1
Hypoxia	0	2	4
Ventricular extrasystoles	0	0	1

Table S3. Summary of Adverse Events by Severity, SOC and PT.

						Group	
						Best practice	Hyperbaric oxygen (HBO)
					Count	Count	Count
Severity	SO		PT				
Mild	SOC	Cardiac disorder	PT	Atrial fibrillation	0	1	0
		Cardiac disorders	PT	Bradycardia	0	1	0
				Chest pain	0	0	1
				Ventricular extrasystoles	0	0	1
		Ear and labyrinth disorders	PT	Ear pain	0	1	1
		General disorders and administration site conditions	PT	Fever	0	0	1
		Infections and infestations	PT	Abscess	0	1	0
				Fungal infection	0	0	1
		Metabolism and nutrition disorders	PT	Diabetes mellitus	0	2	0
		Nervous system disorders	PT	Headache	0	0	1
		Psychiatric disorders	PT	Claustrophobia	0	0	1
		Respiratory, thoracic and mediastinal disorders	PT	Cough	0	1	0

		Vascular disorders	PT	Hypotension	0	1	0
Moderate	SOC	Cardiac disorder	PT	Atrial fibrillation	0	1	0
		Infections and infestaions	PT	Bacteremia	0	1	0
		Psychiatric disorders	PT	Anxiety	0	1	0
				Depression	0	0	1
		Respiratory, thoracic and mediastinal disorders	PT	Hypoxia	0	2	4
		Vascular disorders	PT	Hypotension	0	0	1
Severe	SO	Cardiac disorders	PT	Cardiogenic	0	0	1

Table S4. Overview of Serious Adverse Events

	Group			
	Best practice		Hyperbaric oxygen (HBO)	
	Count	Sum	Count	Sum
SAEFREQ	6	14	6	9

Table S5. Summary of Serious Adverse Events by System Organ Class and Preferred Term

				Group	
				Best practice Count	Hyperbaric oxygen (HBO) Count
SOC	Cardiac disorders	PT	Cardiac arrest	1	0
			Cardiogenic Shock	0	1
	Infections and infestations	PT	Pneumonia	4	0
	Respiratory, thoracic and mediastinal disorders	PT	Hypoxia	7	7
			Pneumothorax	1	0
	Vascular disorders	PT	Hypotension	1	1

Table S6. Summary of Serious Adverse Events by Severity, SOC and PT.

						Group	
						Best practice	Hyperbaric oxygen (HBO)
						Count	Count
Severity	Mild	SOC	Infections and infestations	PT	Pneumonia	1	0
			Respiratory, thoracic and mediastinal disorders	PT	Hypoxia	0	1
					Pneumothorax	1	0
			Vascular disorders	PT	Hypotension	0	1
	Moderate	SOC	Infections and infestations	PT	Pneumonia	3	0
			Respiratory, thoracic and mediastinal disorders	PT	Hypoxia	6	3
	Severe	SOC	Cardiac disorders	PT	Cardiac arrest	1	0
					Cardiogenic	0	1
			Respiratory, thoracic and mediastinal disorders	PT	Hypoxia	1	3
			Vascular disorders	PT	Hypotension	1	0
